# The tiny Hairless protein from *Apis mellifera*: a potent antagonist of Notch signaling in *Drosophila melanogaster*

**DOI:** 10.1186/1471-2148-8-175

**Published:** 2008-06-17

**Authors:** Dieter Maier, Anna X Chen, Anette Preiss, Manuela Ketelhut

**Affiliations:** 1 Universität Hohenheim, Institut für Genetik (240), Garbenstr. 30, 70599 Stuttgart, Germany

## Abstract

**Background:**

The Notch signaling pathway is fundamental to the regulation of many cell fate decisions in eumetazoans. Not surprisingly, members of this pathway are highly conserved even between vertebrates and invertebrates. There is one notable exception, Hairless, which acts as a general Notch antagonist in *Drosophila*. Hairless silences Notch target genes by assembling a repressor complex together with Suppressor of Hairless [Su(H)] and the co-repressors Groucho (Gro) and C-terminal binding protein (CtBP). Now with the availability of genomic databases, presumptive Hairless homologues are predicted, however only in insect species. To further our understanding of Hairless structure and function, we have cloned the *Hairless *gene from *Apis mellifera *(*A.m.H*) and characterized its functional conservation in *Drosophila*.

**Results:**

The *Apis *Hairless protein is only one third of the size of the *Drosophila *orthologue. Interestingly, the defined Suppressor of Hairless binding domain is interrupted by a nonconserved spacer sequence and the N-terminal motif is sufficient for binding. In contrast to *Apis *Hairless, the *Drosophila *orthologue contains a large acidic domain and we provide experimental evidence that this acidic domain is necessary to silence Hairless activity in vivo. Despite the dramatic size differences, *Apis *Hairless binds to the *Drosophila *Hairless interactors Su(H), Gro, CtBP and Pros26.4. Hence, *Apis *Hairless assembles a repressor complex with *Drosophila *components that may have a different topology. Nevertheless, *Apis *Hairless is sufficient to repress the Notch target gene *vestigial *in *Drosophila*. Moreover, it is able to rescue *Hairless *mutant phenotypes, providing in vivo evidence for its function as a bona fide Notch antagonist.

**Conclusion:**

This is the first interspecies-complementation analysis of the Hairless gene. Guided by evolutionary comparisons, we hope to eventually identify all the relevant structural domains and cofactors of Hairless, thereby opening an avenue for further insights into the repressor-complexes that down-regulate Notch signaling also in other, higher eukaryotes.

## Background

Cell to cell communication is essential for development and cellular differentiation of metazoans. The communication is established by signaling pathways that allow information to be sent from one cell to a neighboring cell. This information enables the receiving cell to adopt a different cell fate. One of the best studied signaling pathways that coordinate developmental decisions is the Notch pathway [1-3]. It was first described in the process of lateral inhibition in *Drosophila*: within a cluster of equipotential cells destined to adopt the same cell fate, one cell gains the ability to inhibit adjacent cells to engage differentiation by means of activating Notch. Notch signaling also plays important roles in asymmetric cell divisions that result in differential cell fate decisions [4-6]. Moreover, local Notch activity can induce the formation of developmental boundaries as seen during wing margin formation in *Drosophila *[7-9].

It is not surprising that this fundamental pathway is highly conserved in eumetazoans and is crucial at many different developmental stages in a variety of different tissues [1,2]. The pathway is initiated by the binding of the ligands, Delta or Serrate (Delta-like and Jagged in mammals), presented on one cell to the Notch receptor on the adjacent cells. As a consequence, the intracellular Notch domain is cleaved and migrates into the nucleus, where it forms a transcriptional activator complex by binding, together with co-activators, e.g. Mastermind (Mam), to the transcriptional regulator CSL (**C**SF or RBP-J_κ _in mammals, **S**uppressor of Hairless (Su(H) in *Drosophila *and **L**ag-2 in *Caenorhabditis*) [3]. CSL belongs to the family of rel DNA binding molecules and allows for context specific transcriptional activation of target genes of the Notch signaling pathway [10]. In *Drosophila*, Hairless (H) acts as a general antagonist of this pathway. H binds to Su(H) and, by recruiting the co-repressors Groucho (Gro) and C-terminal binding protein (CtBP), converts Su(H) into a repressor of the Notch target genes [11-14]. In this complex H acts as molecular linker between Su(H) and the co-repressors. Since H retains repressor activity even in the absence of co-repressor binding, it is thought that it impedes formation of the Notch-Su(H)-Mam activator-complex on its own [12].

**Figure 1:**
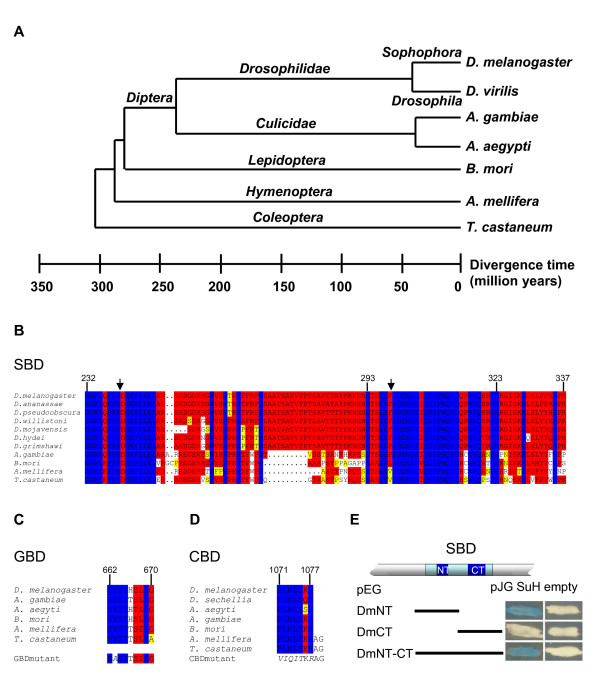
**Conservation of H structural domains in various insects**. A) Evolutionary relationship between insect orders and families, including Drosophilids, Nematocera Diptera, Lepidoptera, Hymenoptera and Coleoptera. Estimated distance is given in million years. B) Alignments of the SBD, (C) the GBD, (D) the CBD of H orthologues from different insects. 13 different Drosophilids were analyzed (see also Additional file 1); only those sequences are depicted that are different from *D. melanogaster *or other closely related species. Both, *A. aegypti *and *A. gambiae *were analyzed; *A. aegypti *is only shown if different from *A. gambiae*. Numbers above the domains correspond to D.m.H amino acid sequence. Below the GBD and CBD, the experimentally introduced mutations are shown. Identical residues are marked in blue; red shows highly conserved and yellow similar residues; dots mark gaps. E) Qualitative analysis of *D. melanogaster *SBD binding to Su(H). Yeast two-hybrid assay to demonstrate interaction between Su(H) [pJG-Su(H)] and SBD or parts thereof. pJG empty vector served as control. Dm-NT-CT overlaps the SBD, Dm-NT includes the N-terminal and Dm-CT the C-terminal portion. Positive interactions are recognized by the blue color caused by transcriptional activation of a lacZ-reporter gene.

Given the high conservation of Notch signaling components, e.g. the human and fly CSL orthologues share approximately 80% identity over large parts [15], one might expect a H homologue to likewise antagonize Notch signaling in mammals. However all attempts from many groups including ours failed so far to identify a vertebrates *H *gene. With the rational that sequences mostly relevant for H function should be conserved over larger evolutionary distance, we started to search for *H *genes in further remote species. Our molecular analysis of the *H *orthologue from the distantly related *Drosophila hydei *species revealed that *H *is indeed a relatively fast evolving gene [16]. Hence, H functional domains may have evolved beyond recognition over time or may be present in different molecules in mammalian species. With more and more genome sequences available, we could identify *H*-like genes in several insect species. From the available genomic sequence projects, we characterized *H *orthologues and found that the *A. mellifera Hairless *gene (*A.m.H*) is a good candidate to investigate H structure and function in more detail. The phylogenetic distance from *Drosophila *to *Apis *is estimated at 250–300 million years and is considerably greater than to *Anopheles *(Fig. 1A) [17]. In contrast to the *Anopheles H *gene, which is larger than the *Drosophila *orthologue *D.m.H*, the *Apis H *gene is much smaller. Moreover, the honeybee brain sequencing project confirmed that *A.m.H *is expressed in honeybee. We cloned the gene from an *Apis mellifera *cDNA library and tested *A.m.H *gene function in *D. melanogaster*. Notably, the orthologous proteins are only 54% identical, and A.m.H is only about 36% of the size of D.m.H. Despite the small size, A.m.H contains the characteristic interaction domains and is able to bind to the *Drosophila *proteins Su(H), Gro, CtBP and Pros26.4 as does D.m.H. Most surprisingly, A.m.H retains functional activity in the fly, where it can rescue *H *loss of function mutant phenotypes. Moreover, when overexpressed, *A.m.H *induces typical H gain of function phenotypes indicative of antagonizing Notch signaling in a variety of tissues and developmental processes. Considering the vast size difference, a repressor complex assembled by A.m.H might have a different topology than that of *Drosophila*. Nevertheless, A.m.H is sufficient to repress the Notch target gene *vestigial *during wing development of *Drosophila*. In contrast to A.m.H, the *Drosophila *H protein contains a large acidic domain and we provide experimental evidence that this acidic domain silences H activity *in vivo*. Finally, our work shows that the Su(H) binding domain previously defined in D.m.H can be separated into two distinct domains, and that the N-terminal domain is sufficient for Su(H) binding. In summary, our work provides evidence for the in vivo function of *Apis Hairless *as a bona fide antagonist of Notch signaling. This evolutionary comparison may help us to eventually identify all the relevant structural domains and cofactors of Hairless, thereby furthering our understanding of the repressor-complexes that down-regulate Notch signaling.

## Results

### Established structural domains of H are well conserved in insect evolution

The *H *gene encodes a general antagonist of the Notch signaling pathway in *D. melanogaster*, where it plays a central role in repressing Notch target genes [18]. There is ample genetic evidence showing that *H *is involved in manifold developmental processes in *Drosophila*. So far it remains open, whether this reflects solely its central role in Notch signaling or whether H is involved in other pathways as well. H is a novel protein that is quite large and may contain more than the already established functional domains. A comparison with the orthologues from *D. hydei *(D.h.H) and *A. gambiae *(A.g.H) identified several conserved domains of presumed functional significance [11,16]. Experimental analysis of *D. hydei *showed that the D.h.H protein with 1158 residues is somewhat larger than the D.m.H orthologue that spans 1077 amino acids [16,19,20]. The A.g.H is even larger and comprises approximately 1300 residues based on an in silico prediction (see Additional file 1). Meanwhile several additional genome sequences have been published. The available databases show that *H *orthologues are present in all Drosophilids (see Additional file 1) and several other dipterans, as well as in other insect orders including Lepidoptera, Hymenoptera and Coleoptera that are considerably further diverged (see Additional file 1). However, to date we could not detect clear H orthologues in species others than insect species, even when using the most highly conserved H domains (see below). The phylogenetic distance between Nematocera dipterans, like *A. gambiae *and *A. aegypti*, and the Cyclorrapha to which Drosophilidae belong, is about 200 – 250 millions years, whereas about 250 – 300 million years are estimated between dipterans and the other insect orders [17] (Fig. 1A). A comparison of the presumptive H orthologues reveals a surprisingly high degree of divergence and highlights conserved domains all the more. These domains characterize the H protein and its function. They include the Su(H) binding domain (SBD), the Gro binding domain (GBD) and, at the very C-terminus a binding sequence for CtBP (CBD) that are remarkably well conserved (Fig. 1B–D). The SBD maps to D.m.H residues 232 to 337, a region that is nearly identical in all Drosophilids (Fig. 1B; see Additional file 1) [16]. However, in further diverged insects including *Anopheles *the SBD is split by a less well conserved stretch (Fig. 1B; see Additional file 1). The GBD has been mapped to nine residues in *D. melanogaster *[11,12]. It is extremely well conserved in the insect H orthologues (Fig. 1C). The CBD is nearly invariant (Fig. 1D).

The structure of the SBD in the further diverged insects led us to address whether the entire region is needed for Su(H) binding (Fig. 1E). In fact we found that the N-terminal portion (NT, L^171 ^to S^270^) was sufficient for Su(H) binding, in agreement with earlier results [21] and bound as well as the entire SBD (NT-CT, L^171 ^to H^357^). In contrast, the C-terminal portion (CT, R^267 ^to H^357^) did not bind to Su(H) (Fig. 1E).

### The H orthologue from *Apis mellifera*

**Figure 2:**
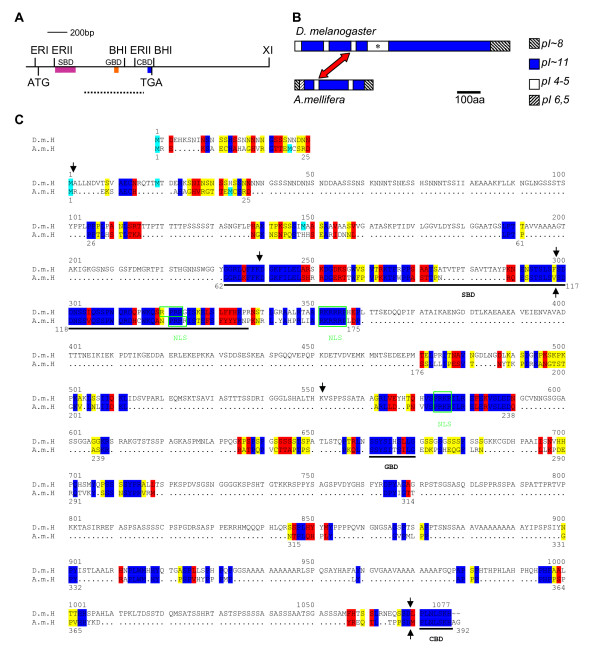
**The H orthologue of *Apis mellifera***. A) Schematic of the A.m.H gene. Location of SBD, GBD and CBD is shown. The dashed line below represents the fragment used to screen the *Apis *cDNA library and to generate fusion-protein for antisera production, respectively. Relevant restriction sites are indicated above the map. ERI = *Eco *RI, ERII = *Eco *RII, BHI = *Bam *HI, XI = *Xho *I. B) pI composition of the H orthologues. The red arrow indicates the acidic domain inside the SBD. Asterisk: acidic domain absent from A.m.H. C) Alignment of the orthologous H proteins from *D. melanogaster *(above, D.m.H) and *A. mellifera *(below, A.m.H). Start codons are highlighted in cyan; in D.m.H the second is the major cap-dependent start site, whereas the third is the internal ribosome entry site [20]. The alignment above shows the start region if the first methionine is ignored. SBD, GBD and CBD are underlined. Nuclear localization consensus sequences (NLS) are boxed in green. Black arrows: position of introns. Blue: identical residues, red: highly similar residues, yellow: similar residues. Black dots represent gaps.

To further our understanding of H function, we analyzed the *Apis mellifera H *gene (*A.m.H*) in more detail. Since there was neither a good annotation of the *A.m.H *gene nor a complete EST sequence available, we cloned *A.m.H *from an *Apis *cDNA library. However, the largest clone missed the start codon by 22 bases as predicted from an incomplete EST-sequence (see Methods). For subsequent in vivo analyses, we extended this cDNA clone by in vitro mutagenesis (Fig. 2A).

The *A.m.H *cDNA spans a total of 2726 bp; it contains two introns at positions identical to *D.m.H *(Fig. 2C). The cDNA has an open reading frame of 392 codons, which is only ~36% of D.m.H. Accordingly, the calculated molecular weight of A.m.H protein is ~44.5 kDa, whereas that of D.m.H is ~110 kDa.

Despite its small size, A.m.H contains the characteristic H protein domains, i.e. the SBD, the GBD and the CBD, and they are well conserved (Figs 1, 2). Three nuclear localization signals are predicted in D.m.H; two are identical in A.m.H, the third is slightly variant (Fig. 2C). D.m.H is a highly basic protein with an isoelectric point (pI) of 10.4; A.m.H is likewise basic with a pI of 10.85. Both proteins contain acidic stretches, however, only the one located within the SBD is conserved (red arrow in Fig. 2B). Unlike D.m.H, the honeybee H protein does not contain the large acidic domain downstream of the SBD (Fig. 2B, asterisk). Using the standard parameters of the BESTFIT program, the two orthologues share 54% identity. Under relaxed conditions that allow an overall alignment, the two protein sequences are 70% similar and 63% identical (see Methods).

### A.m.H protein recapitulates D.m.H protein-protein interactions

The three H domains SBD, GBD and CBD serve as binding sites for the proteins Su(H), Gro and CtBP, respectively. In addition, the C-terminal half of D.m.H was shown to bind to the N-terminal half of Pros26.4, which is one of six AAA-ATPases that form the base of the 19S proteasome regulatory subunit [22].

**Figure 3:**
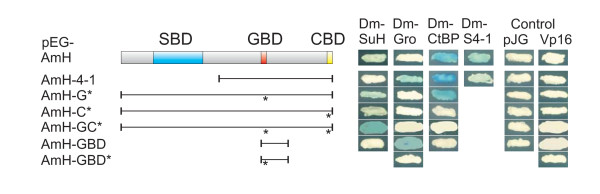
**A.m.H interacts with D.m.H partner proteins**. Protein-protein interactions were tested with the yeast two-hybrid assay. A.m.H wild type and mutant constructs are depicted schematically and were provided in pEG vector that contains the LexA-DNA binding domain. AmH 4-1, N-terminal truncation; A.m.HG*, GBD mutant; A.m.HC*, CBD mutant; A.m.HGC*, double mutant; GBD, Gro-binding domain only; GBD*, mutant construct. Dm-Su(H) and Dm-S4-1 were in pJG; Dm-Gro and Dm-CtBP in VP16 vectors. Empty vectors served as controls. Blue color denotes positive interactions as it reflects activation of the *lacZ*-reporter gene.

The most central feature of H activity is the binding to Su(H). Hence, we first tested whether A.m.H is able to bind to D.m.Su(H). This was not certain since the A.m.H SBD shows just 79% similarity and 72% identity to the *Drosophila *SBD (Fig. 1B). However, full length A.m.H bound to D.m.Su(H), whereas a N-terminally truncated protein that lacks the SBD, A.m.H 4-1 did not (Fig. 3).

The GBDs of A.m.H and D.m.H are 80% identical and 90% similar (Fig. 1C). However, binding of full length A.m.H protein to D.m.Gro protein was not seen in the yeast two-hybrid assay (Fig. 3). This was unsurprising, since full length D.m.H binds rather weakly to Gro, whereas it binds very strongly to a short peptide containing the GBD [12]. Likewise, strong binding was observed with a corresponding small peptide (S^262 ^to P^309^) spanning the A.m.H GBD, as well as with the N-terminally truncated A.m.H 4-1 construct (Fig. 3). The specificity of the interaction was confirmed by a point mutation within the A.m.H GBD: an exchange of Tyrosine 264 to Alanine was sufficient to completely abrogate binding to Gro (Fig. 3), just like the corresponding mutation in *D. melanogaster *[12]. The weak binding of Gro to full length H proteins from either species may perhaps result from the three dimensional structure of H which then must be likewise retained by the tiny A.m.H protein.

The CBD of honeybee and fly H orthologues are identical (Fig. 1D). Therefore, the observed strong interaction between A.m.H and *D. melanogaster *CtBP was expected (Fig. 3). Mutation of A.m.H CBD* completely eliminated binding to D.m.CtBP (Fig. 3) just like the respective D.m.H mutation [12], confirming the specificity the A.m.H CBD. Finally, we tested interaction of A.m.H with Pros26.4. As shown in Fig. 3, the full length A.m.H as well as the N-terminally truncated A.m.H 4-1 both bound to the N-terminal part of Pros26.4 (Dm-S4-1), just like D.m.H. The Pros26.4 binding domain in D.m.H maps roughly between the GBD and the CBD [21]. Interestingly, this region of A.m.H is only 21% of the D.m.H size and contains very few similarities. This comparison will aid to identify the relevant sequences involved in this interaction.

### Activity of A.m.H in *Drosophila melanogaster*

**Figure 4:**
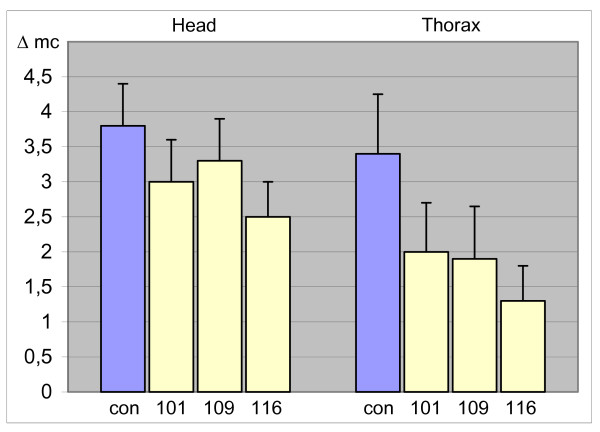
**A.m.H rescues *Drosophila H *mutant phenotypes**. Three different hs-AmH lines (101, 109 and 116) were crossed with *H*^*P8 *^mutant flies at ambient temperature and the number of missing macrochaete (Δmc) on head and thorax scored in comparison to their siblings that had not obtained a transgene copy (purple bar, con). Error bars show standard deviation.

Mutations in the *H *gene are haplo-insufficient in *Drosophila *resulting in a dominant phenotype with reduced numbers of macro- and microchaete and gaps in wing veins [23-25]. Bristle loss can be rescued to about 75% of the wild type numbers by a *D.m.H *gene under the control of a heat shock promoter (hs) at ambient temperature [26,27]. We investigated whether A.m.H can replace D.m.H function in *H*^*P8 *^null mutants. Three independent hs-AmH lines were analyzed. Indeed, a rescue of total macrochaete-loss of 36% was achieved. Moreover, the strongest line restored 62% of the macrochaete on the notum (Fig. 4; Table 1). These data indicate that A.m.H can largely replace D.m.H activity despite the dramatic structural difference between the two proteins.

**Table 1:** Rescue of *H *mutant phenotypes by *A.m.H*

lines	n = number of analyzed flies	a) Rescue mc on head	b) Rescue mc on notum	c) Average rescue *
*yw; + / H*^*P8*^	n = 100			
hs AmH101	n = 24	21%	41%	31%
hs AmH109	n = 33	13%	44%	28%
hs AmH116	n = 45	34%	62%	47%
average		24%	50%	36%

a) Rescue of loss of macrochaete (mc) on the head by hs AmH in %

b) Rescue of loss of macrochaete (mc) on the notum by hs AmH in %

c) Average rescue of loss of macrochaete on head and notum by hs AmH in %.

* student's t test: P_0 _< 0.001.

**Figure 5:**
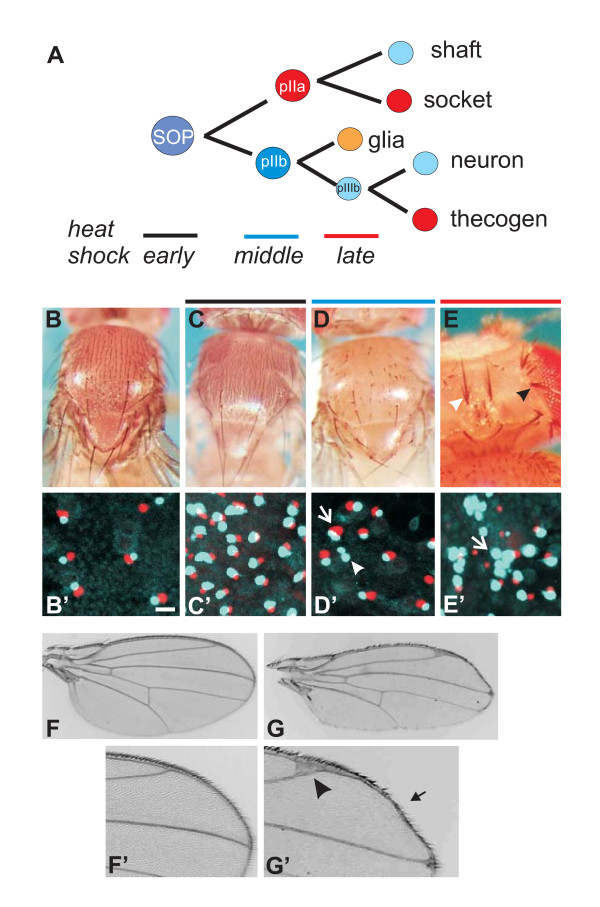
**A.m.H overexpression antagonizes Notch signaling in the process of bristle and wing development**. A) Scheme of bristle organ development. After being singled out from a proneural cluster by lateral inhibition, a sensory organ precursor (SOP) divides unequally to produce two daughter cells (pIIa, pIIb), destined for outer and inner cell fate, respectively. pIIa gives rise to shaft and socket cells, and pIIb eventually to neuron and thecogen. A Notch signal is required for proper designation of cells depicted in red color. B) Thorax of a wild type fly. Note typical arrangement of macrochaete and regular spacing of microchaete, each consisting of a shaft and a socket. B') The underlying cell pair consists of a thecogen (red) and a neuron (light blue). Size bar; 10 μm. C) Early heat induction (black dash; about 5 days after egg deposition) of hs-AmH during larval to pupal transition interferes with lateral inhibition. As a result ectopic SOPs form, each giving rise to a complete bristle organ. Accordingly, additional thecogen/neuron pairs are detected inside (C'; size as in B'). The resultant flies are furry compared to the wild type. D) Later heat shock (blue dash; about 5 days 18 hours after egg deposition) leads to a transformation of outer pIIa to inner pIIb cell fate. Accordingly, neither shafts nor sockets develop, and the flies have bald patches. D') As expected, additional inner cell pairs of thecogen (red) and neuron (light blue) develop (arrow). Moreover, a transformation of thecogen to neuron is observed, giving rise to a neuron doublet (arrowhead). Same size as in B'. E) Heat shock at an even later phase (red dash; about 6 days after egg deposition) causes socket to shaft transformation most easily seen on the head. Consequently, two shafts arise from a bristle organ (black arrowhead). A partial transformation of the socket gives the appearance of a triple shaft (white arrowhead). E') Transformation of inner cell types, thecogen to neuron, is observed as well. In extreme cases, all four bristle cells are transformed to neurons (light blue), giving rise a neuron quadruple (arrow). Size is as in B'. F) The wing of a control fly shows the five longitudinal veins that end thinly at the margin (see enlargement F'). Mechano- and chemosensory bristles cover the anterior, and hairs the posterior wing margin in a regular pattern (anterior is up). G) Heat induction of hs-AmH affects the veins, now ending in typical deltas (arrowhead; see enlargement G'), and the bristles as described above, leaving the impression of a 'sloppy' margin (arrow). In addition, the wing is elongated along the proximo-distal axis, giving it a more lanceolate overall appearance.

Overexpression of H causes phenotypes opposite of the *H *loss of function mutants owing to impairment of Notch signaling. Dependent on the time point of H overexpression (Fig. 5A), ectopic bristles (primarily microchaete), bristle loss or shaft duplications are observed [27,28]. Ectopic bristles form from extra sensory organ precursors that arise when Notch-mediated lateral inhibition is blocked. Indeed, furry flies are generated if hs-AmH is induced during late third larval instar to early pupal stages (Fig. 5B, C). These ectopic sense organs contain the full complement of cells, since we find the corresponding neurons and thecogens in pupal nota (Fig. 5B', C'). The outer bristle organ is derived from the pIIa daughter cell, which is generated by a Notch dependent unequal division of the sensory organ precursor cell (Fig. 5A). Repressing this step during midpupal stages causes a transformation to inner cell fates at the expense of outer cell fates (pIIa to pIIb; Fig. 5A, D) [6,28]. Accordingly, we find bald patches on the outside (Fig. 5D) and pair-wise duplication of inner cell types (neuron plus thecogen) (Fig. 5D'). Finally, Notch signaling is required to differentiate the socket from the shaft cell and the thecogen from the neuron. If these late steps are impeded by overexpression of A.m.H, a socket to shaft transformation (Fig. 5E) and a thecogen to neuron transformation (Fig. 5D', E') is the consequence. Overall, overexpression of A.m.H has the same effects on bristle development as that of D.m.H [26,27]. In addition to the bristle phenotypes, wing phenotypes similar to those of hs-DmH were observed (Fig. 5F, G) [27]. These include irregularities in the bristle pattern along the wing margin, as well as thickened veins that frequently end in broadened deltas (Fig. 5G). In addition to these well characterized phenotypes, we noted an elongation of the entire wing along the proximo-distal axis giving the wings a lanceolate appearance (Fig. 5F, G).

### Tissue specific repression of Notch signaling in *Drosophila melanogaster *by Apis H

**Figure 6:**
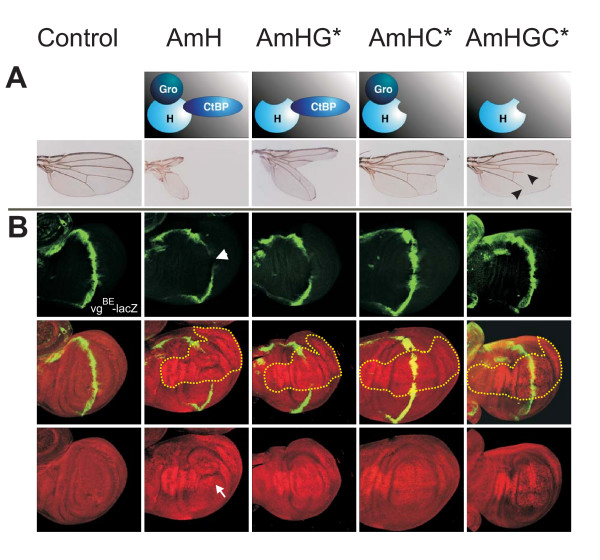
**A.m.H recruits *Drosophila *co-repressorsand downregulates transcription of the Notch target gene *vestigial***. A) Wild type and mutant A.m.H constructs that are deficient in binding Gro, CtBP or both as schematically depicted, were overexpressed in the central wing blade using the omb-Gal4 driver line. Overexpression of these constructs results in deep wing incisions, ranging from nearly complete loss of the wing blade to large notches. Note shortening of longitudinals L4 and L5 upon overexpression of UAS-AmHGC* which cannot recruit any co-repressor (arrowheads). Control flies are omb-Gal4 × UAS-lacZ. Wings are from female flies reared in parallel at 25°C. B) Wild type and mutant A.m.H constructs as depicted above were overexpressed in the central wing domain with omb-Gal4 at 25°C. Expression of the *vg*^*BE*^*-lacZ *reporter gene was determined by anti-beta-galactosidase staining (green). The control disc (omb-Gal4; *vg*^*BE*^*-lacZ*) shows the typical expression in a thin strip of cells along the dorso-ventral boundary (green). Ectopic expression of A.m.H (red nuclear staining) completely represses *vg*-transcription (arrowhead) within the A.m.H overexpression domain (outlined with yellow dots). Lack of Gro co-repressor binding (AmHG*) interferes only little with *vg*-repression, whereas CtBP is clearly more important for H repressor activity (AmHC* and AmHGC*). Expression of A.m.H protein was monitored with antisera (red nuclear staining). Note disturbed wing disc morphology of omb-Gal4>UAS-AmH animals (arrow).

**Figure 7:**
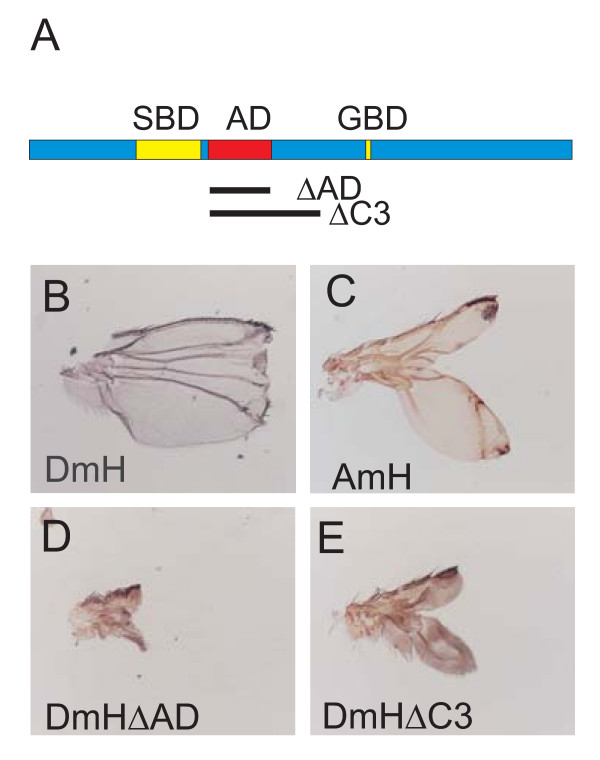
**The acidic domain in D.m.H attenuates H repressor activity**. A) Position of the acidic domain (AD) downstream of the SBD in D.m.H protein. Two deletion constructs (ΔAD, ΔC3) were generated lacking the indicated parts. B) Overexpression of full length D.m.H in the central domain of the wing disc (omb-Gal4>UAS-FLH) results in a truncated wing with a defective distal wing margin. C) In comparison, A.m.H overexpression causes a deep incision, deleting most of the wing blade. D) Overexpression of D.m.H lacking just the acidic domain (omb-Gal4>UAS-HΔAD) causes a much stronger phenotype: the wing is barely detectable, mainly the hinge region remains. E) A similar, albeit less extreme phenotype is observed when D.m.H without the C3 domain is overexpressed (omb-Gal4>UAS-HΔC3).

Notch signaling is involved in a plethora of developmental processes; a well studied example is the formation of the wing margin. During larval life, the presumptive wing margin is established in the wing imaginal disc by activation of the Notch pathway along a dorso-ventral boundary. A number of Notch target genes are subsequently activated including *wingless, vestigial *and *cut*, that are critical for patterning and outgrowth of the wing as well as the specification of wing marginal cells [9,29-31]. If Notch activity is down-regulated in the respective cells, adult wings are incised as exemplified in heterozygous *Notch *mutants [25]. Likewise, wing margin defects develop if *D.m.H *is overexpressed in a spatially and temporally regulated manner in larval wing discs due to a local down-regulation of Notch activity [12,31,32]. Using the Gal4-UAS system [33], *A.m.H *was locally overexpressed using the omb-Gal4 driver line that drives expression in the central part of larval wing discs. This caused extremely deep incisions in the adult wing, and the central wing blade was completely absent (Fig. 6A). Quite surprisingly, A.m.H is able to convert Su(H) into a repressor even more efficiently as D.m.H in the context of wing margin formation (Fig. 7). In *Drosophila*, this conversion is largely dependent on the co-repressors Gro and CtBP [12]. Accordingly, mutant UAS-AmH constructs defective in either binding of Gro (UAS-AmHG*), of CtBP (UAS-AmHC*), or both (UAS-AmHGC*) (Fig. 3) were overexpressed. For each construct at least three independent lines were analyzed that gave similar results. Although we cannot exclude quantitative differences in the expression levels of individual lines, the phenotypic differences were very consistent. UAS-AmHG* caused a less extreme wing nicking than full length A.m.H (Fig. 6A), albeit the phenotype was still very strong and clearly stronger than obtained with D.m.H. Much weaker phenotypes were obtained upon overexpression of AmHC* and even weaker with AmHGC* (Fig. 6A), in accordance with the idea that both co-repressors are required for H repressor activity [12,34]. As already noted for D.m.H, CtBP seems to be the more important co-repressor since loss of CtBP binding reduces H activity more strongly than loss of Gro binding. Notably, overexpression of AmHGC* resulted in shortened longitudinal veins L4 and L5 (Fig. 6A) like DmHGC* [12]. This phenotype is very similar to the dominant wing phenotype of H mutants, raising the possibility of dominant negative effects. Interestingly the AmHGC* double mutant still retains notable repressor activity as seen by the incisions of the wing margin that are caused by its overexpression (Fig. 6A). Hence, A.m.H is able to antagonize Notch signaling independent of co-repressors Gro and CtBP as was noticed before with the *Drosophila *orthologue [12]. Because the small size of A.m.H leaves little room for additional binding domains, it seems not very likely that other, yet unidentified co-repressors confer this repressor activity. Perhaps, some Notch target genes require co-repressors for full silencing whereas others like *vg *do not. In the latter cases, H may directly interfere with the recruitment of intracellular Notch or Mam to the activation complex.

### *Apis *H represses transcription of vestigial in *D. melanogaster*

*vestigial (vg) *is one of the Notch target genes that is activated along the dorso-ventral boundary in the wing imaginal disc and that is important for boundary formation and wing growth [30]. Notch signals activate *vg *expression via the *vg *boundary enhancer (vg^BE^) that contains a Su(H) binding site [29]. The activity of this element is restricted to the dorso-ventral boundary by the Su(H)-Notch activation complex [29] and is repressed in adjacent cells by the Su(H)-H co-repressor complex [12].

Transgenic flies carrying a *lacZ *reporter gene under the control of vg^BE ^(vg^BE^-lacZ) [29] were used to study the ability of A.m.H to regulate *D.m*.*vg *transcription dependent on *Drosophila *co-repressors Gro and CtBP (Fig. 6B). Cells overexpressing A.m.H protein were labeled with anti-A.m.H antibodies (Fig. 6B). Compared to the normal expression of the *lacZ*-reporter gene, beta-galactosidase was almost completely absent in areas, where full length A.m.H was overexpressed, reflecting its repressor activity on the vg^BE ^enhancer element. At the same time, the presumptive wing blade was notably distorted (Fig. 6B), which is typical of full length D.m.H overexpression [12]. Overexpression of AmHG*, resulted in a likewise inhibition of vg^BE^-lacZ expression, however, the wing disc had only little or no morphological defects (Fig. 6B). In contrast, mutation of the CBD interfered strongly with A.m.H repressor activity, since overexpression of AmHC* caused only a small gap in the vg^BE^-lacZ pattern (Fig. 6B). In the absence of co-repressor binding (AmHGC*), no or very little down-regulation of vg^BE^-lacZ was observed (Fig. 6B).

### The acidic domain in *Drosophila *H attenuates its repressor activity

Since the rescue capacities of the heat shock A.m.H constructs were reduced compared to similar D.m.H constructs, we were surprised by the strong wing defects of the omb-Gal4>UAS-AmH flies (see Fig. 6A). These phenotypes were much stronger than those effected by a likewise overexpression of UAS-DmH (Fig. 7B, C) [12]. What could be the reason? Despite the much smaller size of A.m.H, the overall organization of the two orthologues is similar. There is, however, a striking difference with regard to a large acidic domain (AD) in D.m.H just C-terminal of the SBD (Fig. 7A), which is completely absent from A.m.H (Fig. 2B). Our earlier work suggested that the acidic domain might attenuate H repressor activity in *Drosophila *[27]. To strengthen this hypothesis, we generated the D.m.H UAS-HΔC3 and UAS-HΔAD constructs both lacking the acidic domain (Fig. 7A). Both constructs were overexpressed using the omb-Gal4 driver line. As shown in Fig. 7D and 7E, D.m.H protein lacking the acidic domain is much more potent than full length D.m.H or A.m.H. The resultant wings are barely recognized as such since only the hinge region is present (HΔAD; Fig. 7D) or little of the wing blade remains (HΔC3; Fig. 7E). The fact that HΔC3 gives milder phenotypes than HΔAD suggests that regions important for normal H repressor activity are lost in this larger deletion as well. These findings strongly support the notion that the acidic domain antagonizes the repressor activity of D.m.H. Hence, the stronger overexpression phenotypes obtained with A.m.H in the wing nicely fit this hypothesis because of the missing acidic stretch in the *Apis *H orthologue.

**Figure 8:**
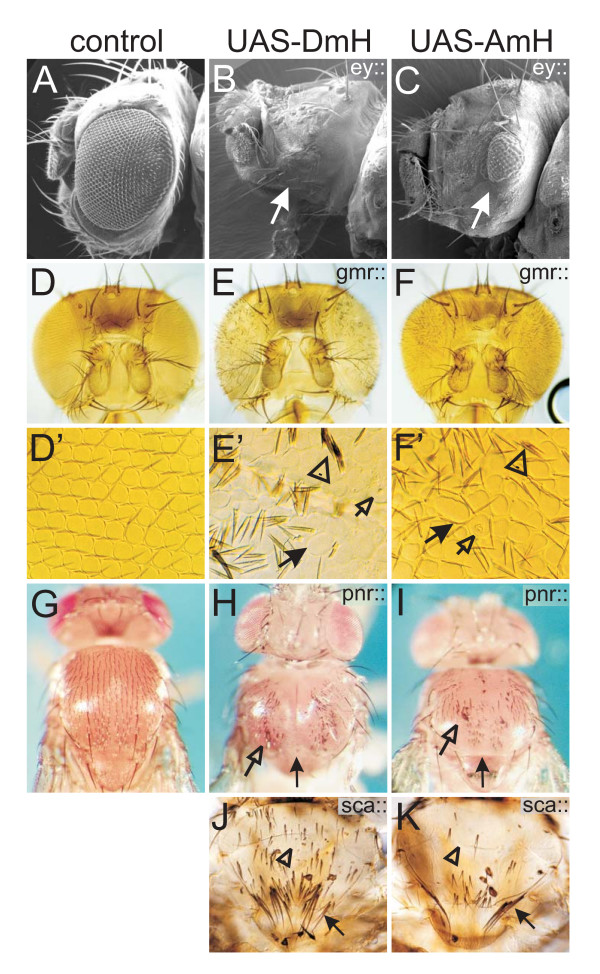
**A.m.H antagonizes different Notch-dependent processes during eye and thorax development**. D.m.H (central panel, UAS-DmH) and A.m.H (right panel; UAS-AmH) full length constructs were overexpressed in a spatially and temporally controlled manner using the Gal4-UAS system. Controls (left panel) are derived from crosses of the same Gal4-line with UAS-lacZ. A-C) Ey-Gal4 drives expression of H in the developing eye disc. As a consequence of impeded cell proliferation and increased cell death, adult eyes are small or completely absent (arrows). D-F) Gmr-Gal4 drives expression of H behind the morphogenetic furrow, which interferes with the process of photoreceptor and cone cell fate determination, respectively. As a consequence of misspecification of cells and cell death, adult eyes are smaller and have a rough appearance. D'-F') Enlargements show that the regular architecture of ommatidia (see D') is disturbed due to fusions and disarrangement (arrows in E' and F'). Interommatidial bristles (arrowheads in E' and F') are duplicated or lacking. Some ommatidia show signs of cell death (open arrows in E' and F'). G-I) Pnr-Gal4 drives H expression in the central region of the thorax anlagen, which interferes with Notch-mediated dorsal closure. In addition to a smaller size due to impeded cell proliferation and increased cell death, the thorax has a marked cleft (arrow). Note also bristle loss and duplications (open arrow). J, K) Sca-Gal4 drives H expression in proneural clusters, resulting in bristle loss (open arrow), additional and split bristles (arrow), as outlined in Fig. 5.

**Figure 9:**
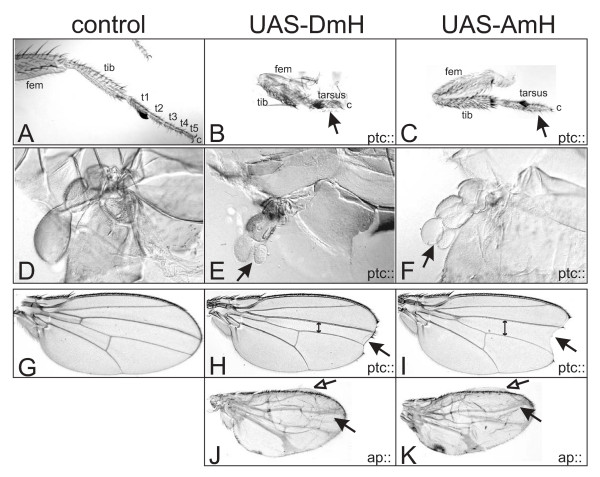
**A.m.H antagonizes many different Notch-dependent processes during appendage development**. D.m.H (central panel, UAS-DmH) and A.m.H (right panel, UAS-AmH) full length constructs were overexpressed in a spatially and temporally controlled manner using the Gal4-UAS system. Controls (left panel) are derived from crosses of the same Gal4-line with UAS-lacZ. A-C) Notch signaling is required for proximo-distal patterning of the leg, notably the formation of segmental joints. Ptc-Gal4 drives H expression in all larval discs along the antero-posterior border. One consequence is the repression of Notch signaling during leg development, causing fusion of the otherwise separated tarsomeres (t1–t5, arrow in B and C) of the tarsus. Moreover, femur (fem) and tibia (tib) are not well separated. D-F) Overexpression of D.m.H and A.m.H in the ptc pattern leads to split halteres (arrow in E and F). G-K) During wing development, Notch is required for outgrowth of the wing blade, margin formation and establishment and refinement of the veins. G) Control wing. H, I) The distance between the third and fourth longitudinal wing veins is conspicuously reduced upon overexpression of D.m.H along the antero-posterior border (ptc-Gal4). In addition, a gap is seen in the distal wing margin (arrow). I) The former phenotype is less pronounced using A.m.H (double arrow; compare with H). Hence, the wing incision appears larger (arrow). J, K) Ap-Gal4 drives H expression in the dorsal compartment, resulting in broadened veins (arrows) and smaller dorsal surface causing blisters (open arrows).

Notch is required for the correct development of a multitude of tissues owing to its involvement in cell growth, cell death and cell differentiation [1,2,35]. Accordingly, overexpression of D.m.H interferes amongst others with growth and patterning of the eye, the wing, the leg and other appendages, dorsal closure of the thorax and bristle specification on the entire body [28-32,36-38]. A.m.H shows the same variety of overexpression phenotypes indicating that it acts as a bona fide Notch antagonist in all known Notch dependent processes (see Figs 8, 9). We note, however, that in most tissues, overexpression of A.m.H resulted in weaker phenotypes than that of D.m.H suggesting a specific role of the acidic domain of D.m.H during wing development.

## Discussion

### Comparison of A.m.H and D.m.H peptide sequence

Most strikingly, A.m.H is roughly a third of the size of D.m.H. A closer look at the D.m.H sequence reveals many poly-residues stretches, notably poly-Alanine, poly-Serine and poly-Asparagine, which are absent in A.m.H, challenging their functional importance. Moreover, A.m.H lacks the large acidic domain that attenuates D.m.H repressor activity. Accordingly, overexpression of A.m.H causes more severe defects than D.m.H, most notably during wing margin formation. However, deletion of the D.m.H acidic domain results in an even more active protein (Fig. 7), demonstrating two important points: on one hand, the acidic domain is a necessary functional domain of Hairless in *Drosophila *that is absent in the honeybee. On the other hand, A.m.H is less active in *Drosophila *compared to the endogenous D.m.H protein.

We noted one additional larger conserved domain of unknown function upstream of GBD (Fig. 2C). It contains a highly conserved nuclear localization signal, which may be the primary reason for the conservation. Other than that, there are only a few very small conserved sequence stretches between the H orthologues from *Apis *and fly and they are not conserved between *Drosophila *and *Tribolium *or *Anopheles *(see Additional file 1). Hence it seems unlikely that they are of functional relevance. With respect to the phylogenetic distance and great divergence of the *Apis H *gene, the remaining activity of A.m.H is quite remarkable. It is able to rescue the dominant *H*^*P*8 ^mutant phenotype and can reproduce if overexpressed largely all phenotypes that are obtained by the overexpression of D.m.H protein (Figs 4, 5, 6; Figs 8, 9). Hence, A.m.H acts as a bone fide antagonist of Notch signaling in *Drosophila*. Since H is a multi-functional protein, this can only be possible if A.m.H protein is able to interact genetically and physically with the components provided by *Drosophila*.

### A.m.H assembles a functional repression complex on Notch target genes using *Drosophila *components

Interaction of A.m.H with *Drosophila *proteins was tested with two powerful approaches, the yeast two-hybrid assay and even more convincingly, a direct in vivo assay in the fly. So far, we know of four direct H interaction partners in *Drosophila*, Su(H), Gro, CtBP and Pros26.4 [11-13,21,22]. Recent data provide evidence that H assembles a repressor complex on Notch target genes by linking Su(H) with the two co-repressors Gro and CtBP. Binding of H to the Pros26.4 subunit of the proteasome is unrelated to Notch signaling [11,12]. However, it reduces H protein stability. Therefore, Pros26.4 indirectly plays a positive role in the Notch signaling pathway [22]. Our data show that A.m.H physically interacts with any of these four *Drosophila *proteins in an in vitro assay. Moreover, we show that relevant mutations in the A.m.H GBD and CBD eliminate binding to the respective co-repressors. These data highlight the importance of the mutated residues for the binding to the respective partner. Interestingly, the A.m.H double mutant retains repressor activity independent of the co-repressors, suggesting that it may interfere with the assembly of the Su(H)-Notch-Mam activator complex. A similar intrinsic repressor activity was already observed for the corresponding D.m.H*GC mutant [12]. This intrinsic repressor activity has been conserved through considerable evolutionary time and we hope to be able to localize the responsible domain by further comparison and to understand the underlying molecular mechanisms. Taken all together *Apis H *is a mini-gene in comparison to the *Drosophila *orthologue. Interestingly this small gene mimics *H *function in *Drosophila *almost completely. This was very surprising since H executes its functions solely through protein-protein interactions. From the functional complementation we conclude that A.m.H must be able to assemble an effective repressor complex together with the *Drosophila *proteins Su(H), Gro and CtBP. However, whereas D.m.H is rather big with roughly 120 kDa and hence provides a sufficiently large surface area for the binding of all three proteins at once, A.m.H. has a predicted size of only about 45 kDa. For example, any of its *Drosophila *partners has a considerably larger molecular weight [12]. Hence, one might expect steric hindrance in a repressor complex containing A.m.H plus *Drosophila *components. Because A.m.H functions well in the fly, it must allow for a topology similar to D.m.H. In this case, the interaction domains SBD, GBD and CBD must be in likewise close proximity in D.m.H, whilst the intervening, non-conserved sequences loop out. A structural analysis of Hairless proteins is required to eventually resolve the conformation of the repressor complex.

### Notch signaling pathway in the honeybee

In this work, we have used A.m.H for a structure-function analysis of fly Hairless. We do not know, whether A.m.H has the same antagonistic role during Notch signaling in the honeybee as in *Drosophila*. Since we cannot genetically manipulate the honeybee in the same way as *Drosophila*, we cannot address this question directly. Instead, we searched the honeybee database for other components of the Notch pathway (see Additional file 2). In fact, we found single orthologues of Notch, Su(H), Gro and CtBP that are extremely well conserved in *Apis*, and one reasonably well conserved Mam orthologue. Moreover, predicted Notch target genes *mγ*, *mβ*, *mβ' *and *mα *that form the honeybee *E(spl)-C *have been already described [39]. We were surprised, however, by the low conservation of *Apis vestigial (vg)*. The A.m.vg protein has a similarity of 63% and an identity of 58% to D.m.vg (see Additional file 2). This is the lowest conservation rate of any Notch pathway component we have looked for. We were curious whether the boundary enhancer, where Su(H) binds to, is present within the presumptive *A.m.vg *gene. In *Drosophila*, this enhancer is located in the first intron and contains a single Su(H) binding site on the minus-strand with the core sequence GTGAGAA [29]. The corresponding intron in the honeybee *vg *gene comprises over 30 kb and more than 20 possible Su(H) target sequences (Genomatrix), three with the identical core sequence on the minus-strand. Taken together, these findings imply that the entire Notch signaling cascade is conserved in *A. mellifera*.

In addition, our data indicate that A.m.H antagonizes Notch signaling in the honeybee also by the assembly of a repression complex consisting of A.m.Su(H) and the co-repressors A.m.Gro and A.m.CtBP. This is based on the high conservation of the three orthologues as well as their binding sites within A.m.H and on the direct protein interactions between A.m.H and the *Drosophila *Su(H), Gro and CtBP proteins (Figs 1, 3; see Additional file 2). The structure of the activation complex from mammals as well as from *C. elegans*, comprising Notch, Mam-like protein and DNA-bound CSL has been elucidated [40,41] and we do not expect it to be much different in fly or honeybee. Differences may arise for target genes such as *E(spl) *or *vg*, which eventually implement Notch signals. The Apis *E(spl) *homologues possess typical Su(H) binding sites in their enhancer-promoters, indicating their importance in the Notch signaling pathway for honeybee development as well [39,42].

### Notch antagonists in higher eukaryotes

Hairless is the general antagonist of the Notch signaling pathway in *Drosophila*. To date, H has no known homologue in higher eukaryotes other than insects, in contrast to the other Notch pathway members that are well conserved from worm and fly to human. Strikingly, the Su(H) protein, which directly binds to H, shares 82% amino acid identity with its mouse orthologue RBP-J_κ _over large protein portions [15]. This is surprising and leads to speculations. For example, it was postulated that H is the counterpart of the Msx2-interacting nuclear target protein (MINT) [43,44]. However, the corresponding *Drosophila *protein is encoded by *split ends (spen) *and has been proposed to integrate information from several different signaling pathways. Recently it has been shown to function also as genetic antagonist of certain Notch dependent processes [45].

## Conclusion

A vertebrate H homologue has not yet been identified based on sequence conservation, presumably due to a high degree of divergence. One experimental approach to eventually identify such a homologue is to analyze the H structure in detail and to characterize important functional domains. The H orthologue from the honeybee will help us in this process. We have shown that A.m.H functions as a bone fide Notch antagonist in the fly despite considerable divergence with regard to size and amino acid sequence.

## Methods

### PCR and cloning strategies

An *Apis mellifera *embryonic cDNA Uni-ZAP XR library [46] was screened with a PCR-probe (see Fig. 2A). Two positive clones were isolated and sequenced. Based on the sequence of the A.m. EST-clone # BB160014B20G05 [47], which covers the N-terminus of a predicted *A.m.H *transcript, our longest isolated cDNA clone was incomplete at its 5' end. The 22 lacking bases were extended using the ExSiteTM PCR-based Site-Directed Mutagenesis Kit (Stratagene). An additional base was added to the lower primer such that the *Eco *RI site provided by the pBluescript vector allowed subsequent in frame cloning in pEG- and pMAL-vectors, respectively. Primer sequences used in this study for in vitro mutagenesis and DNA amplification are available upon request, as are details on the cloning strategies.

### Computer analysis of H orthologues

The *Drosophila melanogaster *gene and protein sequences were accessed in FlyBase [48]. The other *Drosophila *sequences as well as the sequences of *Anopheles gambiae*, *Aedes aegypti*, *Culex pipiens*, *Bombyx mori*, *Tribolium castaneum*, *Apis mellifera*, *Nasonia vitripennis *and *Pediculus humanus corporis *were screened with tblastn service of Flybase. In case of *A. mellifera*, screening was done also with the HUSAR TBlastN2 service of the DKFZ [49,50]. For both databases, we used the *D. melanogaster *protein sequence as search sequences. Similarity and identity scores were calculated using BESTFIT. Because standard conditions only align the best conserved domains, we relaxed the parameters such that the entire protein sequence was aligned (gap weight 1, length weight 1, maximum penalty length 30). Whereas these changes have little influence on identity values of closely related sequences, they give higher scores with less conserved sequences. Multi-alignments were done with PRRN with gap extension penalty 1 and the gap open penalty 9. Further analyses were performed as previously described [39].

### Generation of *A.m.H *and *D.m.H *wild type and mutant constructs

Full length *A.m.H *cDNA was cloned into pUAST [33] generating *UAS-AmH*. Likewise, *hs-AmH *was cloned using pCaSpeR-hs RX8 vector [27]. Mutant constructs were generated with the Quick change XL Site directed mutagenesis kit (Stratagene) according to the manufacturer's protocol. AmHG*: Gro binding site was destroyed by mutating Y264 into A. AmHC*: CBD was modified from PLNLSKH to VIQITKR. AmHGC*: within the mutant construct AmHG*, wild type CBD was replaced by mutant CBD* (Fig. 2A). All changes were sequence verified. The mutant constructs were shuttled into pUAST and pEG vectors, respectively.

Construction of *D. melanogaster Hairless *C3 deletion (R^355 ^to V^564^) was described earlier [27]; it was shuttled into pUAST to yield UAS-DmHΔC3. The AD deletion (E^358 ^to E^465^) was generated by A. Bravo-Patiño. It was likewise shuttled into pUAST (UAS-DmHΔAD).

### Generation and analysis of transgenic flies

All P-element constructs, hs-AmH, UAS-AmH, UAS-AmHG*, UAS-AmHC*, UAS-AmHGC*, UAS-DmHΔAD and UAS-DmHΔC3, were injected into *y*^*1 *^*w*^*1118 *^embryos according to standard protocols and several independent transgenic fly lines were each established; they behaved largely identical in subsequent tests. The results shown are from parallel experiments involving a minimum of three independent lines each and are representative for the respective construct. The obtained phenotypes were non-overlapping. The *H*^*P8 *^null mutant was described earlier [19,51]. Heat shock was given for half hour at 39°C to third instar hs-AmH larvae and early pupae. Overexpression experiments with UAS-lines were performed at 18°C and 25°C, respectively. As driver lines, omb-Gal4, gmr-Gal4, pnr-Gal4, sca-Gal4, ap-Gal4, ey-Gal4 and ptc-Gal4 were used [48]. The vg^BE^-lacZ reporter line [29] was combined with omb-Gal4, and males crossed to UAS-AmH wild type or UAS-AmH mutant virgins. Wing discs of female larvae with the genotype omb-Gal4/X; vg^BE^-lacZ/UAS-AmH* were processed for antibody staining. Control animals were omb-Gal4/X; vg^BE^-lacZ.

### Analysis of protein-protein interactions

Yeast two-hybrid protein interaction assays were performed as previously described using VP16-dCtBP, VP16-Gro, pEG-Gro, pJG-S4-I and pJG-Su(H) [12,22,31]. For bait, *Apis mellifera *constructs pEG-AmH, pEG-AmHG*, pEG-AmHC* and pEG-AmHGC* were used. In addition, the following pEG-constructs were generated: pEG-AmHGBD containing the Gro binding domain (codons S^262 ^to D^309^), pEG-AmHGBD* (Y^264 ^to A mutation, PCR-amplified from AmHG*) and pEG-AmH4-1 (deletion of 172 N-terminal codons). AmH4-1 is an incomplete cDNA clone; it starts with L^173 ^and contains complete C-terminal coding sequences. The *D. melanogaster *Su(H)-binding domain (SBD) was subdivided into DmH-NT (codons L^171 ^to S^279^) and DmH-CT (codons R^267 ^to T^362^) and cloned into pEG vector, respectively. pEG NT-CT (L^171 ^to T^362^) spans both parts. All constructs were sequence confirmed. Expression of the various pEG-constructs was examined with Western blots using the anti LexA antibody (Invitrogen).

### Immuno-histochemistry and phenotypic analyses

A PCR construct spanning A.m.H codons N^137 ^to P^348 ^was cloned into pMAL-C expression vector (New England Biolabs). AmH-MBP fusion protein was expressed in *E. coli *and affinity purified using standard protocols. Polyclonal antisera were from Pineda ABservice (Berlin). Imaginal discs were stained as described before using rat anti-AmH (1:500) and mouse anti-beta-galactosidase (1:20) (developed by J.R. Sanes; obtained from Developmental Studies Hybridoma Bank [DSHB], Department of Biological Science, University of Iowa City, IA 52242). Pupal nota were dissected as described earlier [6,28] and stained with rat anti-elav 7E8A10 and mouse anti-pros MR1A (each 1:10) (developed by G.M. Rubin and C.Q. Doe, respectively; obtained from DSHB).

Secondary antibodies coupled to fluorescein and Cy3 were purchased from Jackson Laboratory. Samples were mounted in Vectashield (Vector Lab) and analyzed on a Zeiss Axioskop linked to a Bio-Rad MRC1024 confocal microscope. Fly body parts were dehydrated in ethanol and mounted in Euparal or Hoyer's medium. Pictures were taken on a Zeiss Axiophot with Nomarsky optics. Pictures of adult flies were taken with a Pixera camera on a Wild 5M stereo-microscope using Pixera Viewfinder 2.0. They were assembled using Corel Photo Paint and Corel Draw software.

### Accession numbers

The *Apis mellifera Hairless *sequence is available from the EMBL Nucleotide Sequence Database under the accession number: AM849041.

## Authors' contributions

DM conceived of the study, acquired and analyzed the data and drafted the manuscript. AXC cloned the DNA-constructs, made transgenic flies and started a first phenotypic analysis. AXC and MK performed the yeast two-hybrid assays. AP helped in the analysis of fly phenotypes, performed pupal notum stainings and helped to draft the manuscript. All authors read and approved the final manuscript.

## Supplementary Material

Additional file 1 The additional file AF1 (pdf format) contains two Figures, Fig. S1 and Fig. S2. Figure S1 shows a multi-species alignment of the orthologous H protein sequences from 13 different Drosophilids. Figure S2 contains a multi-species alignment of orthologous H protein sequences from 9 different insect species, including several dipterans, silk worm, beetle, two different hymenoptera and body louse. Click here for file

Additional file 2 The additional file AF2 (pdf format) contains Figure S3 that shows an alignment of the *Drosophila melanogaster *and the *Apis mellifera *orthologues Notch, Su(H), Gro, CtBP, Mam and Vg. Click here for file
