# Supplementary material for: The tiny Hairless protein from Apis mellifera: a potent antagonist of Notch signaling in Drosophila melanogaster
Source: BMC Evol Biol. 2008 Jun 17;8:175. doi: 10.1186/1471-2148-8-175 (PMC2440387; doi:10.1186/1471-2148-8-175)
Supplement: Additional file 1 — The additional file AF1 (pdf format) contains two Figures, Fig. S1 and Fig. S2. Figure S1 shows a multi-species alignment of the orthologous H protein sequences from 13 different Drosophilids. Figure S2 contains a multi-species alignment of orthologous H protein sequences from 9 different insect species, including several dipterans, silk worm, beetle, two different hymenoptera and body louse. [file 1471-2148-8-175-S1.pdf]

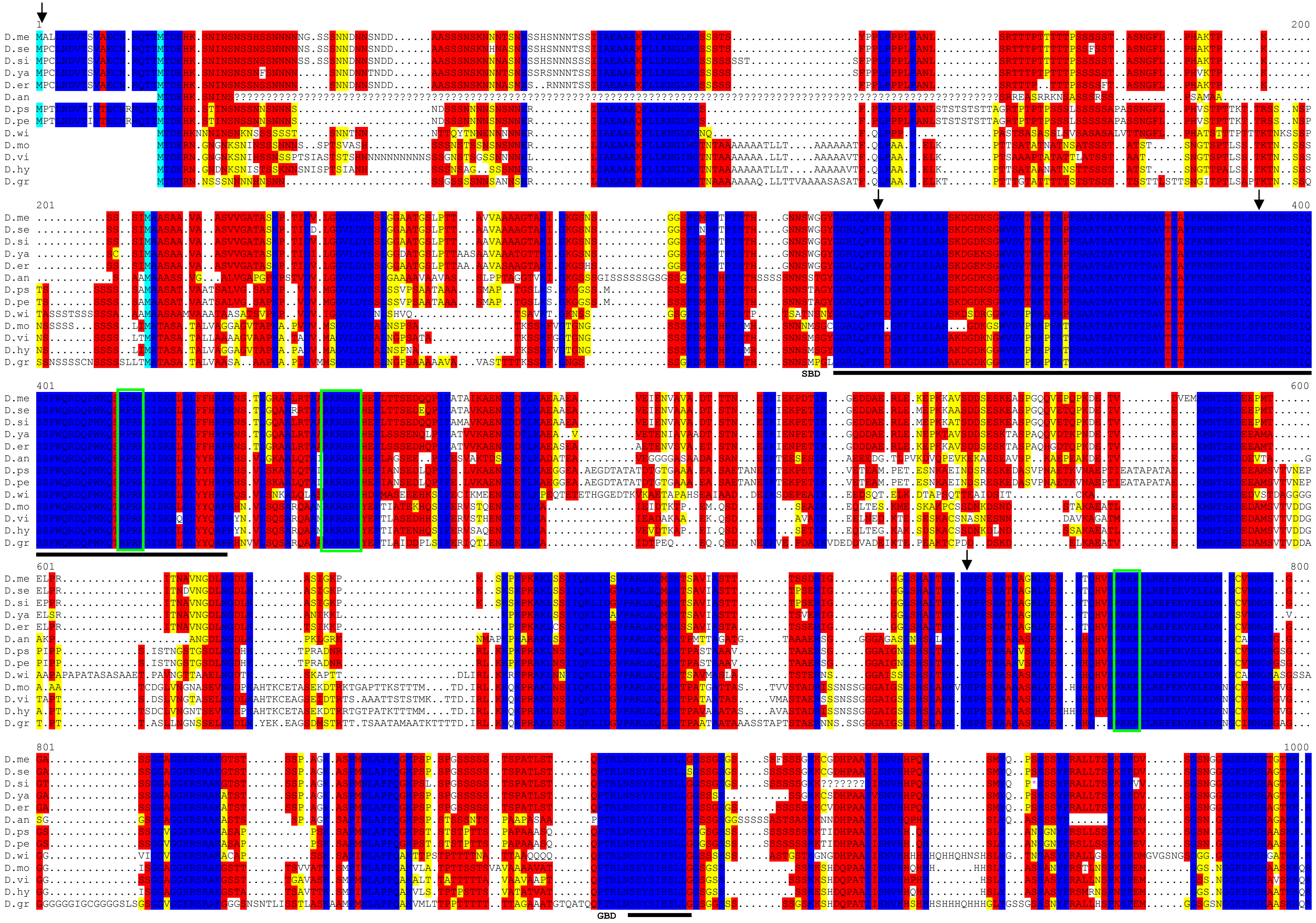

D.me
D.se
D.si
D.ya
D.er
D.an
D.ps
D.pe
D.wi
D.mo
D.vi
D.hy
D.gr
1
200
201
400
401
600
601
800
801
1000
SBD
GBD

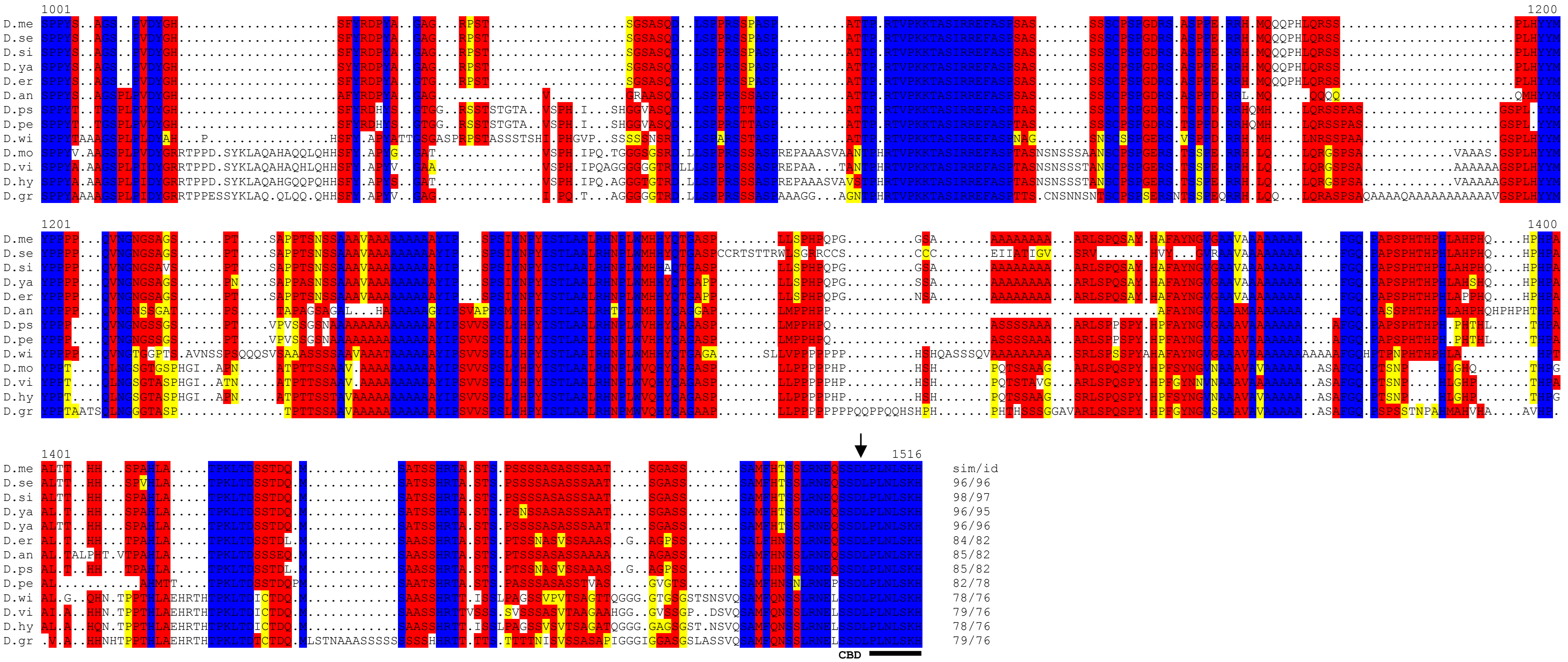

1001
1200
1201
1400
1401
1516
D.me
D.se
D.si
D.ya
D.er
D.an
D.ps
D.pe
D.wi
D.mo
D.vi
D.hy
D.gr
sim/id
96/96
98/97
96/95
96/96
84/82
85/82
85/82
82/78
78/76
79/76
78/76
79/76
CBD

Figure S1

Multi-species alignment of H orthologues from Drosophilids

Hairless protein sequences were predicted from genomic sequences. SBD, GBD and CBD are underlined. H orthologues from Drosophilids contain each four introns at identical positions (arrowheads). Presumptive start methionines are shown in cyan. Green boxes: presumptive nuclear localization signals. Blue, identical amino acids; red, highly similar amino acids; yellow, similar amino acids; dots represent gaps. Similarities and identities with respect to the *D. melanogaster* sequence are given in percent. Sequencing gap in *D. ananassae* (D.an) is shown by question marks. D.me: *D. melanogaster*, D.se: *D. sechellia*, D.si: *D. simulans*, D.ya : *D. yakuba*, D.er : *D. erecta*, D.ps: *D. pseudoobscura*, D.pe: *D. persimilis*, D.wi: *D. willistoni*, D.mo: *D. mojavensis*, D.vi: *D. virilis*, D.hy: *D. hydei*, D.gr: *D. grimshawi*.

The BESTFIT program was used for calculation of similarity and identity scores (gap weight 1, length weight 1, maximum penalty length 30). The multi-alignment was performed with PRRN program. Parameters of multi-alignment were: gap extension penalty 1, gap open penalty 4.

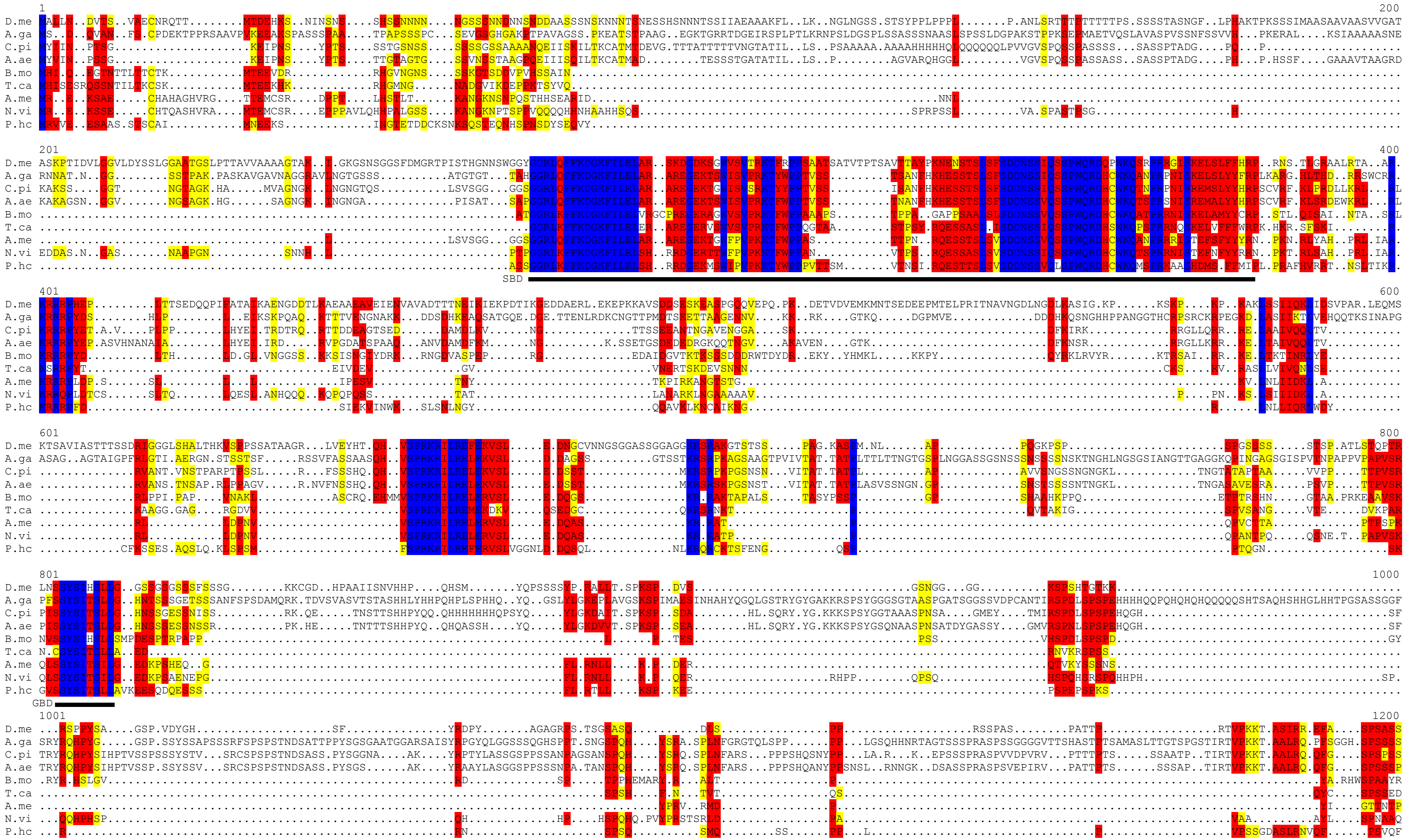

1
200
D.me
A.ga
C.pi
A.ae
B.mo
T.ca
A.me
N.vi
P.hc
201
400
SBD
401
600
601
800
801
1000
GBD
1001
1200

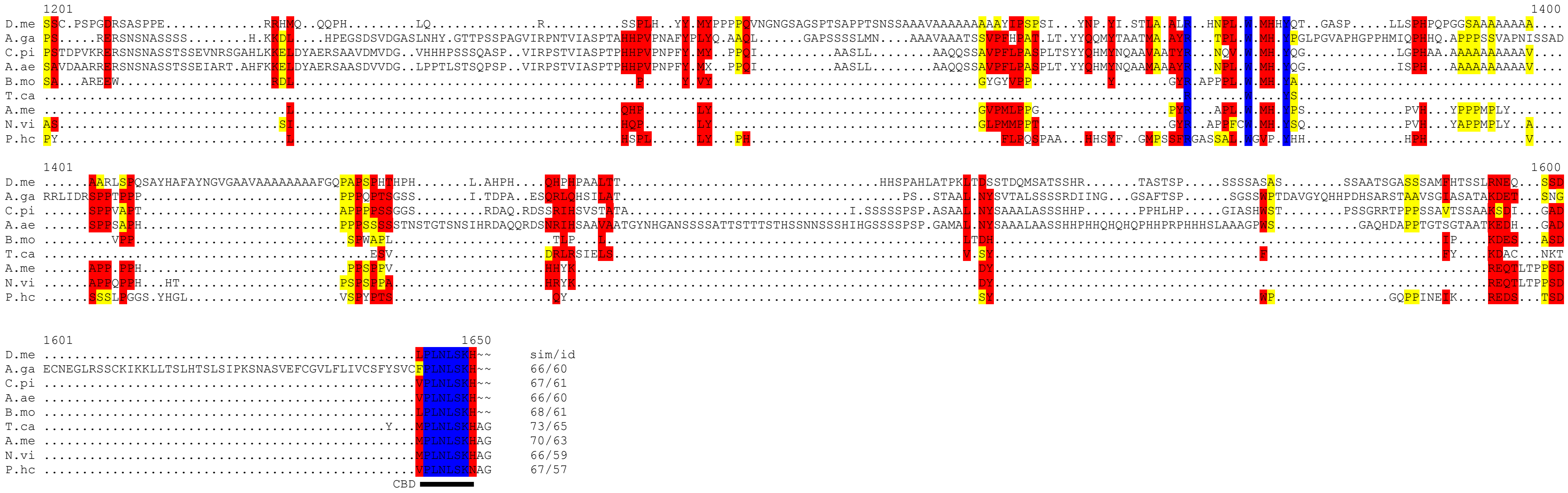

1201
1400
1401
1600
1601
1650
D.me
A.ga
C.pi
A.ae
B.mo
T.ca
A.me
N.vi
P.hc
sim/id
66/60
67/61
66/60
68/61
73/65
70/63
66/59
67/57
CBD

Figure S2

Multi-species alignment of H orthologues from different insects

Comparison of *Drosophila melanogaster (D.me.), Anopheles gambiae (A.ga.), Culex pipiens (C.pi), Aedes aegypti (A.ae), Bombyx mori (B.mo.), Tribolium castaneum (T.ca.), Apis mellifera (A.me.), Nasonia vitripennis (N.vi) and Pediculus humanus corporis (P.hc)* is shown. Protein sequences were predicted from genomic DNA apart from D.m.H and A.m.H. that are based on sequenced cDNAs. SBD, GBD and CBD are underlined. Sequence similarities and identities are given in percent with respect to the D.m.H. Blue, identical amino acids; red, highly similar amino acids; yellow, similar amino acids; dots represent gaps. For programs and parameters, see Figure S1.
