# Supplementary material for: The tiny Hairless protein from Apis mellifera: a potent antagonist of Notch signaling in Drosophila melanogaster
Source: BMC Evol Biol. 2008 Jun 17;8:175. doi: 10.1186/1471-2148-8-175 (PMC2440387; doi:10.1186/1471-2148-8-175)
Supplement: Additional file 2 — The additional file AF2 (pdf format) contains Figure S3 that shows an alignment of the Drosophila melanogaster and the Apis mellifera orthologues Notch, Su(H), Gro, CtBP, Mam and Vg. [file 1471-2148-8-175-S2.pdf]

A) *D. melanogaster* and *A. mellifera* Notch orthologues

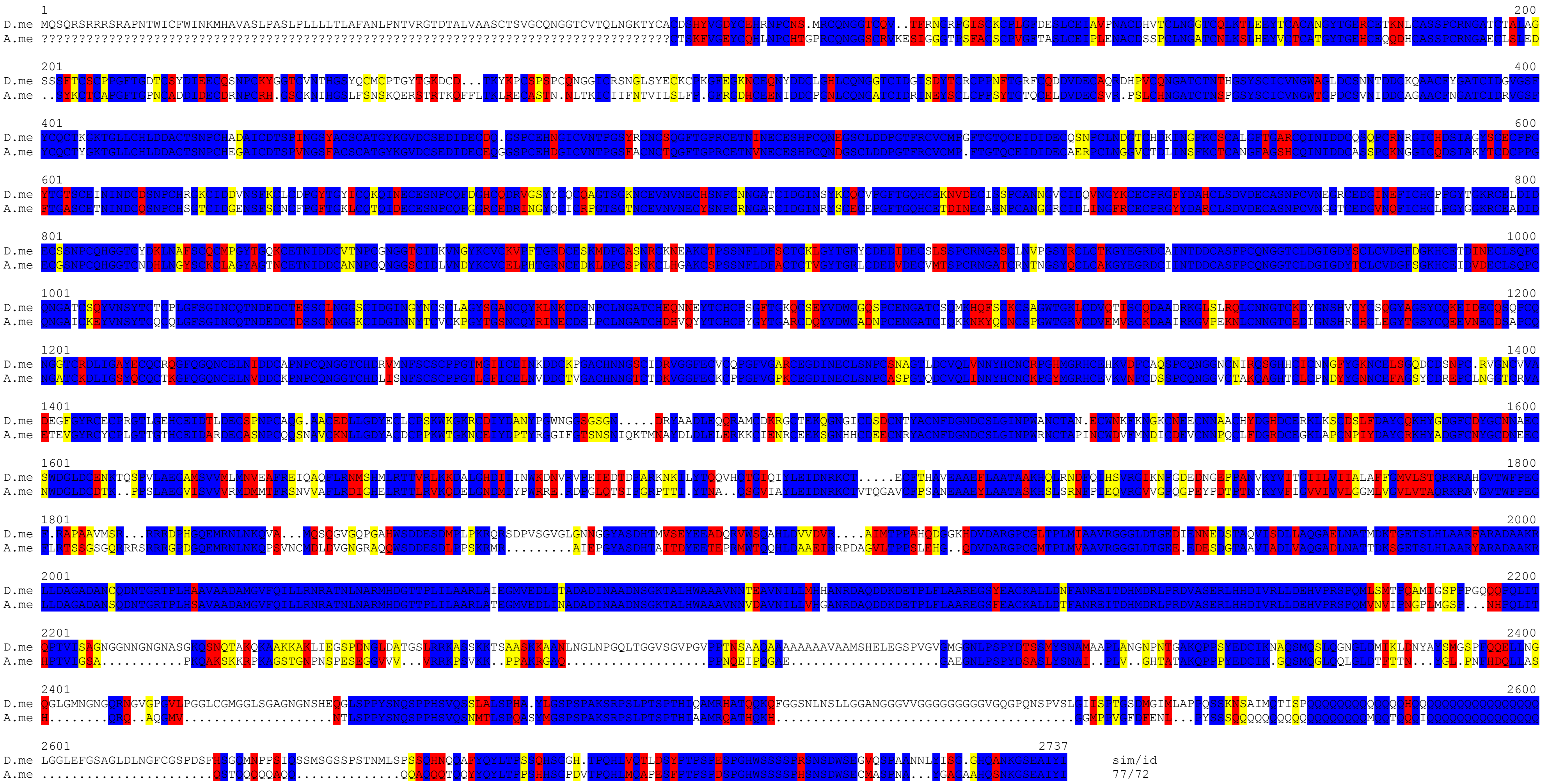

B) *D. melanogaster* and *A. mellifera* Su(H) orthologues

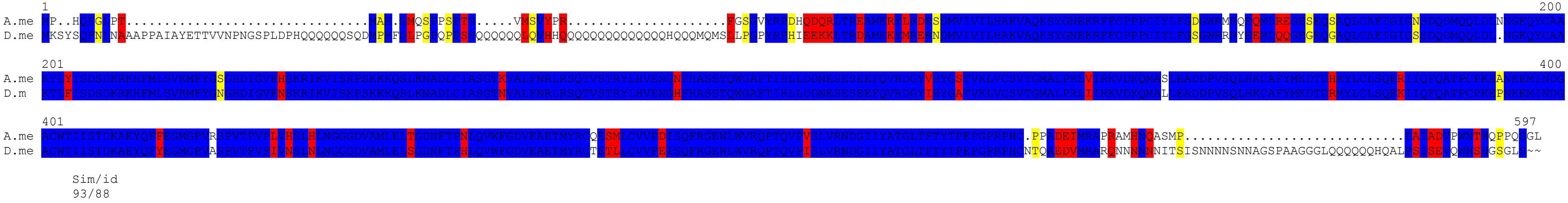

C) *D. melanogaster* and *A. mellifera* Groucho orthologues

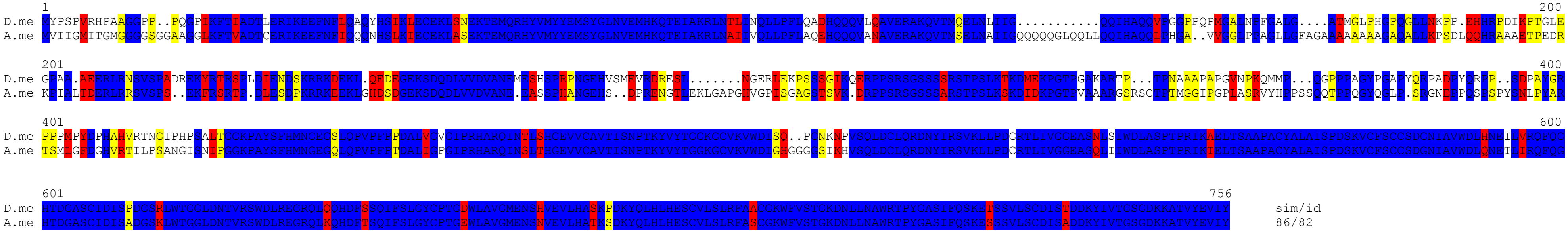

D) *D. melanogaster* and *A. mellifera* CtBP orthologues

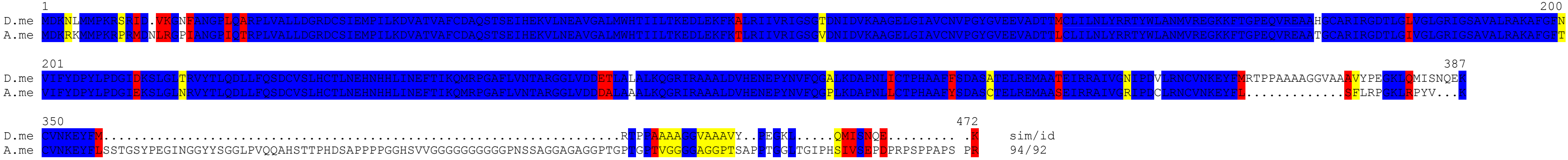

E) *D. melanogaster* and *A. mellifera* Mastermind orthologues

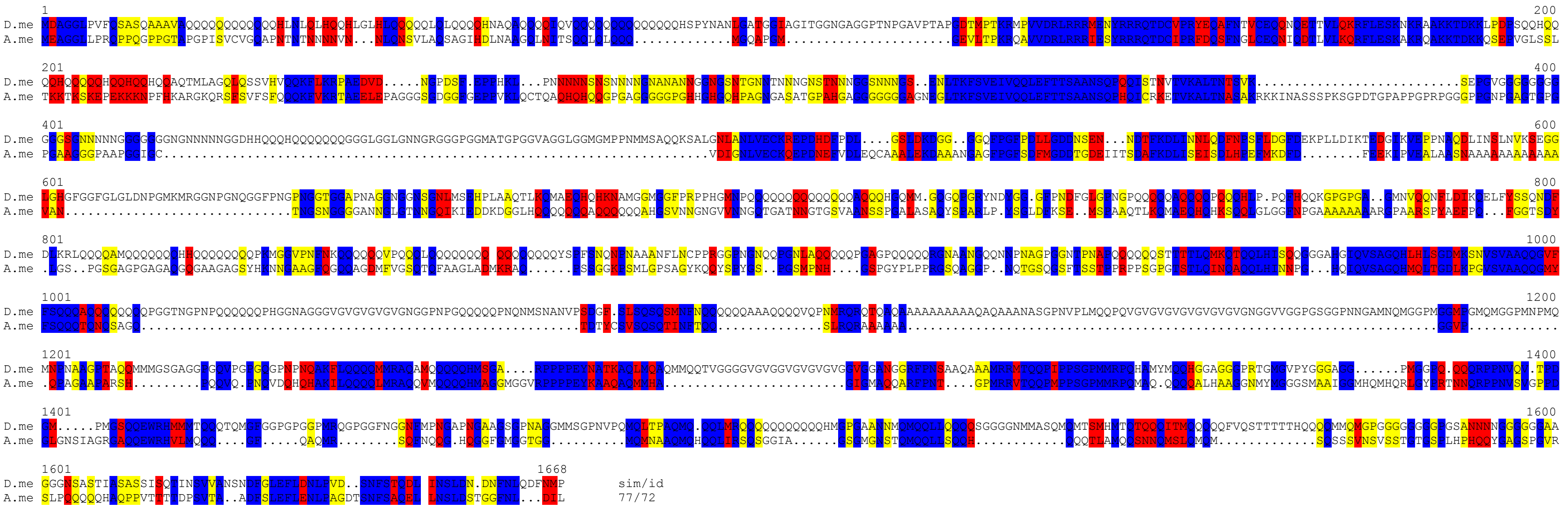

F) *D. melanogaster* and *A. mellifera* Vestigial orthologues

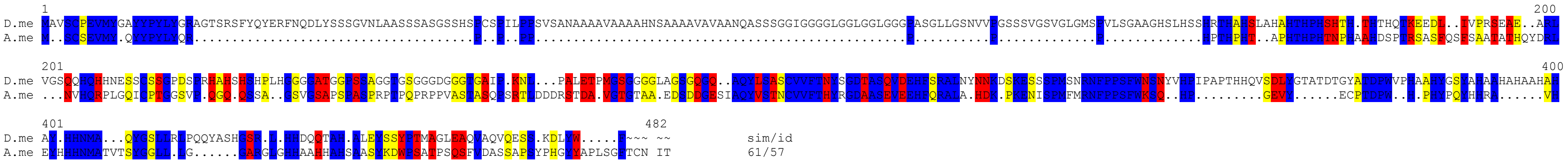

Figure S3

Conservation of Notch pathway components in honeybee.

Single orthologues of Notch, Su(H), Gro, CtBP, Mam and Vg were identified in the honeybee genome. A.m. Protein sequences were deduced from genomic sequence and aligned with the respective *D. melanogaster* protein sequences. Blue, identical amino acids; red, highly similar amino acids; yellow, similar amino acids; dots represent gaps. Sequences similarities and identities are given in percent with respect to *D. melanogaster*. Gap in A.m.N is depicted by question marks.

Similarity and identity scores were calculated using BESTFIT (gap weight 1, length weight 1, maximum penalty length 30). Multi-alignments were done with PRRN with gap extension penalty 1 and the gap open penalty 9, apart for the Vestigial comparison, where gap open penalty 4 was applied.
